# OCCUPATIONAL THERAPY AND NEUROPSYCHOLOGY: INTERDISCIPLINARY COLLABORATION IN CURRICULUM DEVELOPMENT

**DOI:** 10.1093/geroni/igae098.3112

**Published:** 2024-12-31

**Authors:** Theresa Christopher, Jennifer Christopher

**Affiliations:** North Central College, Naperville, Illinois, United States; Jesse Brown VA Medical Center, Chicago, Illinois, United States

## Abstract

Interdisciplinary care models have a positive impact on older adult care (Harris, 2006). This is especially true for people diagnosed with dementia (Galvin et al., 2014). Neuropsychologists and occupational therapists are vital team members in providing care to people diagnosed with dementia. However, there is a lack of research highlighting the collaboration between these two professions. The current authors believe that graduate education is an opportune time to initiate these collaborations. The current poster aims to showcase a partnership between a neuropsychologist and occupational therapy professor. This collaboration resulted in a one-hour lecture provided by a neuropsychologist to masters of occupational therapy students in a geriatric theory course. The lecture outlined the role of the neuropsychologist in differential diagnosis of dementia and the opportunities for interdisciplinary collaboration between occupational therapists and neuropsychologists. The authors believe that this lecture is beneficial to routinely include in standard occupational therapy geriatric curriculum. In the future, the authors hope to conduct a quantitative study to examine the effectiveness of this lecture in promoting students’ understanding of dementia and the partnership between neuropsychology and occupational therapy. References Galvin, J. E., Valois, L., & Zweig, Y. (2014). Collaborative transdisciplinary team approach for dementia care. Neurodegenerative disease management, 4(6), 455-469. doi: 10.2217/nmt.14.47. Harris, B. A. (2006). Interdisciplinary education: What, why, and when? Journal of Physical Therapy Education, 20(2), 3-8.

